# Streptococcus intermedius Pleuropulmonary Disease: A Not So Commonly Seen Radiological Picture

**DOI:** 10.7759/cureus.17385

**Published:** 2021-08-23

**Authors:** Azka Tasleem, Aqsa Mahmood, Rajeev Sharma

**Affiliations:** 1 Internal Medicine, Indiana University Health Ball Memorial Hospital, Muncie, USA

**Keywords:** infection, abscess, empyema, streptococci, infiltrates

## Abstract

A *Streptococcus *(*S.*)* intermedius-*associated pleuropulmonary infection (PPI) is a rare presentation. Cases have been reported in the last decade in which *S. intermedius* was identified in pleural fluid cultures and lung biopsies in patients presenting with complicated pleural effusions and empyema. However, there are data lacking on predisposing factors, mode of dissemination, and treatment strategies for these patients. Moreover, these patients can present with a spectrum of radiographic findings, which can help with prompt diagnosis and effective patient management. However, the radiological findings in this patient and the rapidly worsening clinical symptoms make the diagnosis difficult.

## Introduction

*Streptococcus *(*S.*)* intermedius* is a facultative anaerobe belonging to the S. anginosus group. This group includes three organisms: S. anginosus, S. intermedius, and S. constellatus. S. intermedius is commonly involved in causing abdominal and brain abscesses. S. constellatus causes pulmonary and blood infections, and S. anginosus causes gastrointestinal and genitourinary infections. Streptococcus intermedius rarely is associated with pleuropulmonary infection (PPI), which includes pneumonia, pleural effusion, and empyema [1-2]. Risk factors involve smoking, alcohol abuse, dental illness, chronic obstructive pulmonary disease, malignancy, liver cirrhosis, and diabetes [3]. Through this case report with a literature review, we discuss an acute presentation of S. intermedius necrotizing pneumonia with radiological findings, difficult to distinguish as lung abscess or empyema, and compare it with radiographic findings seen in the few cases diagnosed so far.

## Case presentation

**Figure 1:**
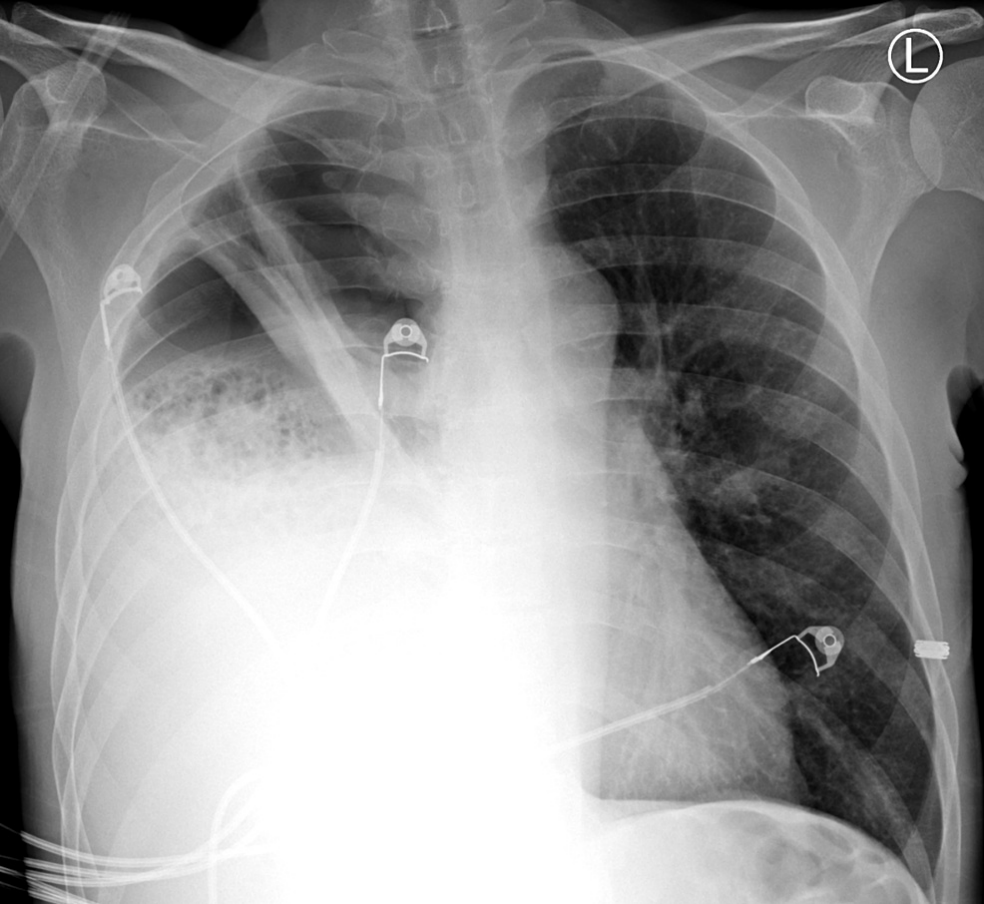
A posteroanterior (PA) chest radiograph showing bubbly lucencies in the right midlung and extensive consolidation

The patient was a 54-year-old male, chronic smoker, who presented with dyspnea and productive cough with yellowish-green sputum for a duration of two weeks. These symptoms were associated with loss of taste, weight loss, and generalized fatigue. He had no known history of aspiration episodes, chest trauma, or surgeries. He initially presented to an urgent care center for the above symptoms with an oxygen saturation percentage in the 80s. He presented to the emergency department on 3 liters/min of oxygen with an oxygen saturation of 96%. Abnormal labs included white cell count (WBC) 26 k/cumm (reference range 3.6-10.6); hemoglobin (Hb) 8.6 gm/dl (reference range 13.4-17); platelets 753 k/cumm (reference range 150-450); and C-reactive protein 26.6 mg/dl (reference range <1). Coronavirus disease 2019 (COVID-19) and influenza swabs were negative. A chest X-ray was done (Figure 1).

**Figure 2:**
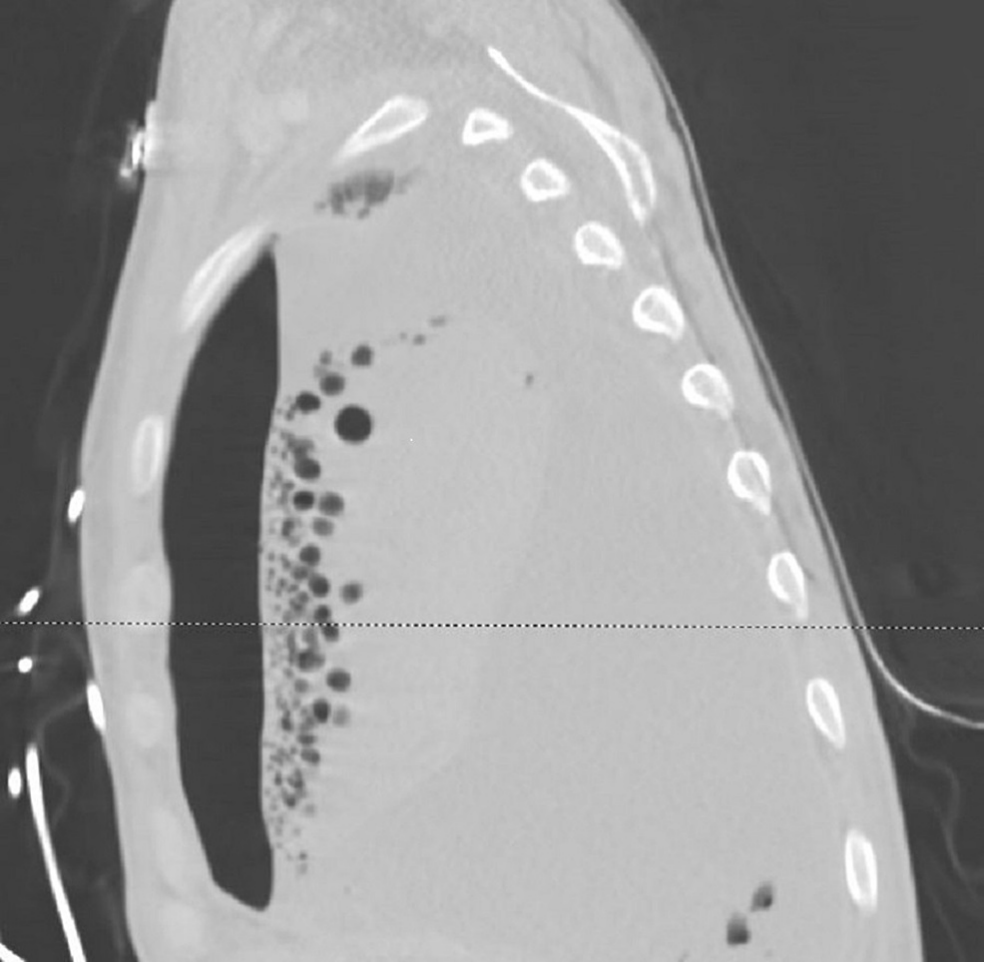
Anteroposterior (AP) view of CT chest showing focal areas of fluid within the pulmonary parenchyma consistent with intraparenchymal abscesses in the right lung

**Figure 3:**
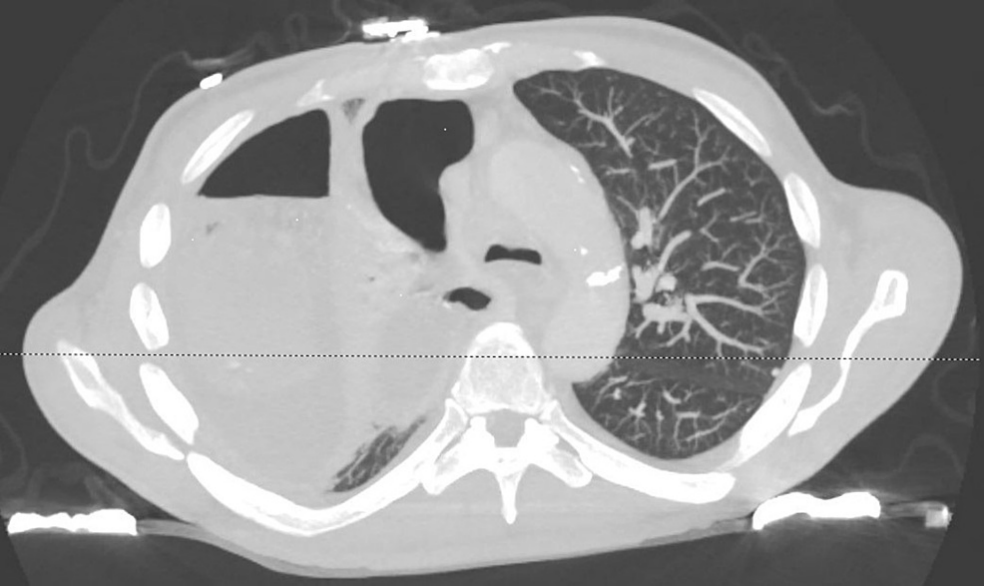
Transverse view of CT chest showing near-complete opacification of the right upper, middle, and lower lobes

**Figure 4:**
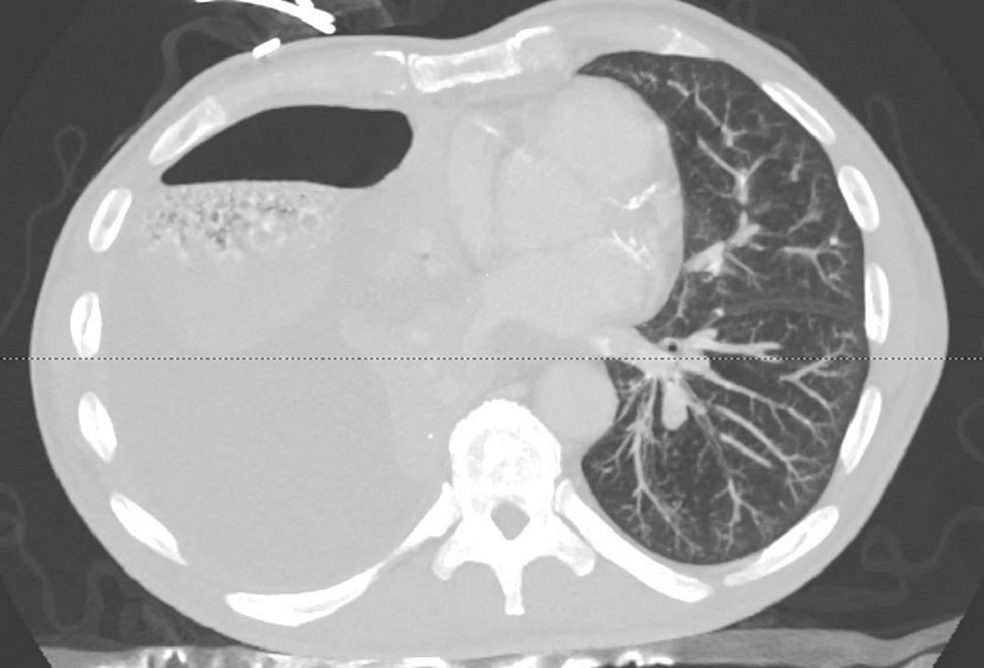
Transverse view of CT chest showing a large right hydropneumothorax with a near-complete filling of the right hemithorax

This was followed by computed tomography of the chest (Figures 2-3), which showed right pleural fluid and gas collection consistent with large empyema filling the majority of the right hemithorax with a mild leftward shift of the mediastinum (Figure 4). Also, hyperdense material within the fluid and near-complete consolidation of the right lung with numerous intraparenchymal abscesses were seen.

The pulmonary department was consulted immediately, and azithromycin and ceftriaxone were given. Cardiothoracic surgery was consulted due to a concern for empyema and the need for drainage. Before evaluation by cardiothoracic surgery, he went into pulseless electrical activity (PEA). Return of spontaneous circulation (ROSC) was achieved in five minutes, and the patient was intubated. He was put on pressor support. The patient underwent a bedside diagnostic bronchoscopy with washing, which showed red and inflamed mucous membranes of right-sided airways. A right-sided apical chest tube was placed, and 1500 ml of reddish-brown fluid was drained. Broncho-alveolar lavage (BAL) fluid was sent for cultures. On day two, bloody secretions were noticed in the nasogastric tube, and there was a suspicion for fistula, neoplasm, or esophageal trauma. Another chest tube was inserted posteriorly later to improve drainage of right lower lobe effusion since the patient was unstable for surgery/decortication. Subsequently, labs in the morning showed white blood cells 65 k/cumm; Hb 6.9 g/dl; lactate 21.5 mmol/l (reference range; 0.5-2.2 mmol/l); anaplastic lymphoma kinase (ALK) 340 units/l (normal range:25-125 units/L), alanine aminotransferase 4240 units/l (normal range: 7-52 units/L), and aspartate aminotransferase, 4161 units/l (reference range: 13-39 units/L). Arterial blood gas analysis showed pH 6.86, HCO3 7 mmol/l, and Pco22 40 mmHg. Antibiotics were switched to broad-spectrum vancomycin and piperacillin-tazobactam. Pleural fluid culture results came back positive for S. intermedius. Testing for blood cultures, urine Streptococcus antigen, Legionella antigen, and Methicillin-resistant Staphylococcus aureus (MRSA) were negative. The patient received fresh frozen plasma for possible disseminated intravascular coagulation and blood transfusions for dropping hemoglobin levels. He continued to worsen and was in profound septic shock on three vasopressors and stress dose steroids to maintain mean arterial pressure greater than 65 mm Hg. The plan was to do video-assisted thoracoscopic surgery (VATS) by cardiothoracic surgery when the patient became hemodynamically stable. However, the patient deteriorated and went into asystole. Eventually, the family intervened and asked to avoid any further efforts to revive the patient. Unfortunately, the patient expired.

## Discussion

*S. intermedius* is a bacterial organism well-known for causing brain and liver abscesses. However, it is a resident of the oral cavity and can be aspirated in patients at risk, causing pleuropulmonary disease. This is a rare phenomenon but there have been cases documented with *S. intermedius* PPI and varied radiological findings. A literature review was performed utilizing PubMed with the keywords “S. intermedius pneumonia” and “pleural effusion,” and articles published from the year 2010 onwards pertinent to *S. intermedius* PPI were reviewed. The emphasis was on the various radiological presentations, diagnostic techniques, and treatment strategies. We reviewed 10 articles in total published from the year 2010 onwards (Table 1).

**Table 1:** Radiological findings, diagnostic technique, and treatment approach in patients diagnosed with Streptococcus (S.) intermedius PPI

ARTICLE	Radiological Findings	Diagnostic Technique	Treatment
Sakurai, 2020 [1]	Right multi-lobular empyema and right iliopsoas abscess	Pleural fluid culture	Doripenem, later deescalated to ampicillin + right small thoracotomy + psoas abscess drainage
Patail, 2020 [4]	Right loculated pleural effusion with passive atelectasis	Pleural fluid culture	VATS
Takahashi, 2019 [2]	Large loculated pleural effusion in the right lung, passive atelectasis	Pleural fluid culture	Ceftriaxone and clindamycin switched to ampicillin-sulbactam later.
Cobo, 2018 [3], 6 patients	Included right hemithorax opacification, nodular lesions, pulmonary abscess, and empyema.	Pleural fluid culture	Ceftriaxone and levofloxacin; Imipenem, ceftriaxone, and Clindamycin (1 case); Levofloxacin, clarithromycin, clindamycin (1 case); Drainage (for all cases)
Kaga, 2017 [5] (multiple empyema)	Chest X-rays taken at the time of admission showed signs of infiltration in both lung fields and suggested bacterial pneumonia; CT head showed a convex lens-shaped area of low attenuation accompanied by a ring of enhancement was present in the left posterior cranial fossa	Blood culture	Clarithromycin + Carbocysteine; had no improvement, later switched to ampicillin and metronidazole
Catalya et al. 2017 [6]	Multiple cavitary lung nodules	Lung biopsy culture	Vancomycin followed by ceftriaxone
Hameed, 2017 [7]	3.5 – 4.5 cm conglomerate nodal mass in the mediastinum compressing the main stem bronchi, Lesions on CT brain.	EBUS culture was negative. Brain biopsy and aspirate culture.	Ceftriaxone, later switched to meropenem. This was followed by amoxicillin-clavulanate for 4 weeks.
Hanoodi, 2016 [8]	Bilateral multi lobular lung infiltrate, loculated effusion	Tissue sample culture	Erythromycin + ciprofloxacin + aztreonam + vancomycin + azithromycin + ceftriaxone Drainage, pleurectomy, decortication
Noguchi et al. 2014 [9]	Consolidation, encapsulated pleural effusion	Consolidation, encapsulated pleural effusion	Meropenem + Thoracentesis+ Left pleurectomy + Chest drainage
Lescan, 2013 [10]	Empyema, Peridural abscess	Peridural space fluid culture	VATS + Systemic antibiotics

The radiological findings seen in the patient population diagnosed with S. intermedius infection broadly include consolidation, loculated pleural effusion, cavitary lesions, nodules, empyema, and lung abscess [1-10]. In our patient, it is difficult to differentiate an empyema vs. an empyema with abscesses or malignancy with superimposed infection. The fatigue and weight loss increase suspicion for malignancy. For diagnostic purposes, negative sputum cultures do not exclude this infection. Instead, pleural fluid culture analysis and lung biopsy are performed if a strong suspicion for S. intermedius PPI is required for definite diagnosis [6].

As per the review of literature, more studies are needed to determine the virulence factors that mediate disease pathogenesis and predilection for specific tissues in S. intermedius pneumonia. Moreover, a few cases have been reported in the literature that mention the presence of multiple abscesses in patients positive for S. intermedius. Hence, looking for abscesses at other sites can help avoid missing any infection source [10].

The ideal treatment approach in these patients includes a combination of both surgical and medical management. Antibiotics employed to treat this infection mostly included vancomycin, levofloxacin, and ceftriaxone. Moreover, surgical decortication has proven to be efficacious in these patients. This case report highlights the fact that with a radiological presentation indicating a loculated pleural effusion, empyema, or lung abscess, physicians should keep “S. intermedius PPI” as a differential, particularly in high-risk individuals. Timely management can reduce mortality and ensure high-value care.

## Conclusions

The radiological findings in this case are unique, in the sense that it is hard to predict if it is just an abscess, empyema, or malignancy with superimposed infection. Also, the patient did not have significant risk factors for S. intermedius infection, thus it makes us think if we still need studies to determine the pathophysiology of the disease and the population at risk. It stresses how rapidly an S. intermedius infection can worsen, hence there should be a high suspicion for this infection whenever clinicians see a similar radiological picture. Early decortication might help with faster recovery; however, unfortunately, this patient was not stable enough to undergo this procedure.

## References

[1] Sakurai M, Nagasawa H, Takeuchi I, Yanagawa Y (2020). A case of an 80-year-old man with empyema and psoas abscess. Case Rep Emerg Med.

[2] Takahashi S, Ishitsuka T, Namatame K, Nakamura M (2019). A false-positive pneumococcal rapid urinary antigen test in Streptococcus intermedius infection. Respirol Case Rep.

[3] Cobo F, Sampedro A, Rodríguez-Granger J, Aliaga-Martínez L, Navarro-Marí JM (2018). Clinical and microbiologic characteristics of pleuro-pulmonary infection due to Streptococcus intermedius. Rev Esp Quimioter.

[4] Patail H, Patail H, Ahmad S (2020). A man in his 30s presenting with shortness of breath and productive cough after a recent pneumonia. Chest.

[5] Kaga A, Higo R, Yoshikawa H (2017). A case of multiple empyema caused by Streptococcus intermedius. Auris Nasus Larynx.

[6] Catalya S, Komal B, Tulpule S, Raoof N, Sen S (2017). Isolated Streptococcus intermedius pulmonary nodules. IDCases.

[7] Hameed S, Singh J, Tricia LB, Machado A, Ruggieri P, Mehta AC (2017). Conglomerate mediastinal mass of a different etiology. Oxf Med Case Reports.

[8] Hannoodi F, Ali I, Sabbagh H, Kumar S (2016). Streptococcus intermedius causing necrotizing pneumonia in an immune competent female: a case report and literature review. Case Rep Pulmonol.

[9] Noguchi S, Yatera K, Kawanami T (2014). Pneumonia and empyema caused by Streptococcus intermedius that shows the diagnostic importance of evaluating the microbiota in the lower respiratory tract. Intern Med.

[10] Lescan M, Lepski G, Steger V, Schlensak C, Walker T (2013). Rapidly progressive paraplegia and pleural empyema: how does that correlate?. Gen Thorac Cardiovasc Surg.

